# Are you ready for retirement? The influence of values on membership in voluntary organizations in midlife and old age

**DOI:** 10.3389/fpsyg.2022.951811

**Published:** 2022-08-23

**Authors:** Julia Sánchez-García, Andrea Vega-Tinoco, Ana I. Gil-Lacruz, Diana C. Mira-Tamayo, Miguel Moya, Marta Gil-Lacruz

**Affiliations:** ^1^Department of Business Management and Organization, University of Zaragoza, Zaragoza, Spain; ^2^Department of Social Psychology, University of Granada, Granada, Spain; ^3^Department of Psychology and Sociology, University of Zaragoza, Zaragoza, Spain

**Keywords:** voluntary membership, values, middle-aged, old age, gender

## Abstract

Membership in voluntary organizations is associated with individual and social benefits. Due to the negative consequences of the global pandemic on older people, and the governmental challenges posed by population aging, voluntary membership is of great importance to society. To effectively promote volunteering among older people, it is necessary to understand the determinants of voluntary membership. This study analyses the influence of individual values—secular/traditional and survival/self-expression–on voluntary membership among European adults (*N* = 31,985). Specifically, it examines which values orient two age groups (middle age: 50–64 and old age: 65–79), as well as men and women toward a certain type of association (Social Awareness; Professional and Political; Education and Leisure; Religion). The sample of 31,985 comprises 60% of adults aged 50–64 and 40% aged 65–79; of which 56% are women and 44% men. The empirical estimation considers different levels of data aggregation: individual, national and welfare system, therefore multilevel analysis is used as an analytical strategy. Individual-level variables from the Integrated Values Survey (2005/09, 2010/14, and 2017/20) and national-level variables (Gini Index and Gross Domestic Product Per Capita) from the World Bank and Eurostat are used. The results indicate that traditional and self-expression values promote membership in voluntary organizations in general more than secular and survival values. However, there are differences according to the type of organization. Furthermore, values are found to moderate the effect of age and gender on voluntary membership.

## Introduction

Retirement can be defined as the exit of a person from the labor force that occurs after middle age (Wang and Shi, 2014). Retirement can be an expected moment in later life (van Solinge and Henkens, 2007). However, very little is known about how adults behave after retirement. There is growing recognition of the potential for retirement to negatively affect the long-term health and well-being of older adults by remaining inactive (Wang and Shi, 2014). With fewer friends over time, with children moving away, and with retirement replacing a phase of life filled with productivity, seniors report a great sense of loneliness and worthlessness (Athavale et al., 2022).

Consequently, it is time to encourage more sustainable mechanisms to address isolation, especially among older people (Payne, 2022). One way to strengthen social inclusion and well-being in old age is membership in voluntary associations (Serrat et al., 2015; Moyano-Díaz and Mendoza-Llanos, 2021; Levasseur et al., 2022), which directly contributes to Sustainable Development Goal 3: *Ensure healthy lives and promote well-being for all at all ages*. In this regard, the empirical evidence shows that membership helps older people to feel more useful and productive, and to enhance their sense of agency and self-esteem (Haslam et al., 2018; Chung et al., 2020; Celdrán et al., 2021; Lam et al., 2021; de Wit et al., 2022).

Continuity Theory explains that the occurrence of voluntary activities in workers can predict the continuation of volunteering when they are retired (Muthuri et al., 2009). However, older people tend to be less likely to volunteer than younger people (Wymer, 1999). Governments, civil society organizations, the private sector and communities should create opportunities to foster active aging after retirement (UNV, 2021). Therefore, it is necessary to identify the motivational factors that lead older people to become volunteer members, to improve recruitment and retention strategies in organizations. Among these motivational factors, the Volunteer Process Model identifies values (Omoto and Snyder, 1995; Wilson, 2012).

Values are considered deep-rooted dispositions that cause people to behave in a certain way (Halman et al., 1993). A well-known classification into two dimensions of Inglehart and Welzel (2005) includes traditional values (emphasizing the importance of religion, deference to authority, and national pride), in contrast to secular-rational values (divorce, abortion, euthanasia, and suicide are considered acceptable), and survival values (emphasis on economic and physical security), in contrast to self-expression values (environmental protection, tolerance of foreigners, homosexuality and gender equality, and civic participation). It seems that membership in a volunteer organization depends to a large extent on the values a person holds (Kearney, 2001). In this sense, the shift from traditional to secular values is associated with a decline in civic participation, while the shift from survival to self-expressive values is associated with an increase in participation (Inglehart, 2017).

According to Modernization Theory, the variation in basic value orientations between generations is due to cultural changes resulting from economic development (Inglehart and Baker, 2000; Inglehart, 2018), with more rational and self-expression values among the younger compared to more traditional and survival values among the oldest. However, it is not known whether the statistical differences between the value orientations of older and younger people affect voluntary membership ratios (Wollebæk and Selle, 2003). Consequently, the main objective of this research is to analyze the individual and contextual determinants of volunteer membership among adults aged 50+, especially those related to values, and in their interaction with age and gender in different types of organizations.

The main contributions of this work are: (1) the analysis of values through two indexes: Traditional/Secular Index and Survival/Self-Expression Index (Inglehart and Welzel, 2005); (2) the comparison between two age groups: middle age (pre-retirement –50–64-), and old age (retirement –65–79) since active aging has to be approached as a dynamic process that requires awareness throughout life; (3) the consideration of gender, to check whether behaviors and values differ between men and women; (4) to study membership in four types of associations: Social Awareness, Professional and Political, Education and Leisure, Religion; (5) considering several European welfare systems (Continental, Nordic, Mediterranean, and Eastern), as well as economic inequality (Gini index) and gross domestic product per capita (GDP). Data from the Integrated Values Survey (IVS; 2005/09, 2010/14, and 2017/20), the World Bank and Eurostat (2005/20) are used. The empirical estimation is carried out considering simultaneously different levels of data aggregation: Individual, National and Welfare Systems.

### Literature review

Voluntary associations are becoming increasingly relevant due to their greater number of members and beneficiaries (Kang, 2014; AbouAssi and An, 2017; Ki and Cho, 2021), as well as their positive effects on the health and well-being of older people (Moyano-Díaz and Mendoza-Llanos, 2021). Voluntary associations are defined as formally organized groups whose members do not receive financial remuneration for their participation (Knoke, 1986). These associations represent activities aimed at various social, cultural, political, professional, industrial, occupational, sports, and religious groups (Tschirhart and Gazley, 2014).

The scientific literature has mainly considered two forms of membership: money, in the form of donations, and time, in the form of volunteering (Kou et al., 2014; Mesch et al., 2021). Regarding the second, being a member of a voluntary organization provides numerous benefits for the volunteer and for the society (Dekker and Feenstra, 2015; Li and Wu, 2019; Lam et al., 2021). In the case of older people, at the individual level, it improves the health, life satisfaction, and happiness (Burr et al., 2021; Vega-Tinoco et al., 2021; de Wit et al., 2022). At the national level, it fosters social solidarity and active aging (Gil-Lacruz M. et al., 2019), which facilitate the sustainability of pension and healthcare systems (Galenkamp and Deeg, 2016), besides generating economic benefits, for example, increasing the levels of Gross Domestic Product (Salamon et al., 2003; Gil-Lacruz and Marcuello, 2013).

Nevertheless, not all membership in a voluntary organization means the same thing. In this sense, Gil-Lacruz A. I. et al. (2019) show that the characteristics and effects of membership in voluntary organizations depend on the type of association in which this labor is performed. For instance, volunteering in social awareness organizations is characterized as instrumental, in which individuals offer to influence public behavior through certain values, and less for the enjoyment of participation itself. As a result, these organizations have a positive impact on life satisfaction, but a negative impact on happiness. Consequently, it is assumed that the motivations and rewards attributed to these associations could differ.

The classical Resource Model of Civic Engagement (Verba et al., 1995) argues that membership in organizations could be explained in terms of mobilizing factors. Niebuur et al. (2018) in a meta-analysis, find that married people and those with high level of education or income are more likely to be members of a voluntary organization. Regarding age, Nichols and Shepherd (2006) indicate that the highest peak of participation is reached at middle age and then declines among the oldest. In contrast, Putnam (2000) find that older people are more likely to volunteer than younger people. However, this result was only found in rich countries, suggesting that this phenomenon could be associated with economic development. Other authors explain that these age differences in volunteering could be due to levels of education and income, the impact of religious tradition on civic attitudes, degree of freedom of expression (Inglehart and Baker, 2000) or the type of welfare system (Salamon et al., 2003).

Overall, the highest proportions of older volunteers are found in Nordic countries, while Mediterranean countries tend to be characterized by lower rates of membership among older people. These findings imply that membership patterns are related to the way societies are structured and how social responsibility is allocated within them (Salamon et al., 2003), revealing, for example, variations in the opportunities to engage in productive activities. This only explains a part of the membership. Sánchez-García et al. (2022), point out that age differences in volunteering depend on the type of activity. The authors show that the 50–64 age group frequently joins professional and political organizations, while the 65–79 age group tends to be involved in religious associations. The question of why they choose to be involved one or the other organization, however, requires further research (Boccacin and Lombi, 2018).

In relation to gender, the scientific literature is inconclusive: some research indicates that men are volunteer members more frequently than women (Einolf, 2011), other that women are more likely to be volunteer members (Women’s Philanthropy Institute, 2022), and some have found no differences (Eagly, 2009). Social Resources Theory developed by Wilson and Musick (1997) argues that these differences between genders could be a consequence of economic resources. People with higher educational level, higher income level and better professional position are more likely to volunteer. In this sense, on average, men have more economic resources and a higher level of education than women (Wiepking et al., 2022), although this is changing in some Western countries, such as Germany (Van Bavel et al., 2018). Other factors that could explain gender differences are time availability (Ariza-Montes et al., 2015), type of organization (Gil-Lacruz A. I. et al., 2019), gender roles (Wemlinger and Berlan, 2016), differences between countries in terms of culture (Spitsyna and Koval, 2022), and national level of gender equality (Sánchez-García et al., 2022). In summary, the decision to become a member of a voluntary association not only depends on sociodemographic factors (Niebuur et al., 2019), it can also depend on altruistic motives, signs of empathy, facilitation, caring, personality, or individual values (Spitsyna and Koval, 2022).

The analysis of the influence of values on membership in an association has been a widespread issue in several disciplines such as psychology or sociology (Dekker and Halman, 2003; Ariza-Montes et al., 2015; Luria et al., 2017). Building on Maslow, Inglehart (2018) argues that older people who experienced wartime in their childhood developed predominantly materialistic value orientations, due to the scarcity of goods. In contrast, younger adults, who were better off in their childhood, developed postmaterialist values, being free to care about ideals such as democracy, human rights, environmental issues, and gender equality.

Putnam (2000) questions this position and describes older people as the “long civic generation,” being more active in associations, and younger people as less civic-minded and more materialistic. He explains that young adults have been brought up to believe that their needs can be satisfied immediately, which has shaped their moral norms and values. Despite these contradictory assumptions, there is no doubt that value orientations generate social change. Therefore, values that are socialized during childhood and adolescence influence adults’ perspectives on participation in voluntary associations (Wollebæk and Selle, 2003).

According to Inglehart (1990, 1997), modernization-economic and political progress-gives rise to long-term intergenerational cultural changes that transform people’s basic values. The first shift as a consequence of modernization involves a transition from traditional values to secular values, associated with industrialization, leading to a lower rate of civic engagement. Societies with traditional values among their members emphasize religion, authoritarian and male-dominated structures, as well as low tolerance for alternative family or gender constructs; whereas societies with secular values exhibit the opposite characteristics (Inglehart, 2017, 2018).

The second change refers to the shift from survival values to self-expression values, the latter related to the emergence of the knowledge society, leading to higher rates of voluntary adherence in general (Dekker and Feenstra, 2015). Inglehart and Baker (2000) explained that self-expression is associated with an elevated trust, tolerance, subjective well-being and political activism that emerges in post-industrial societies with high levels of security. In contrast, survival is associated with low levels of well-being in societies characterized by insecurity, which emphasize physical and economic security above all else, and which feel threatened by foreigners, ethnic diversity, and cultural change.

### The present research

The aim of this study is to assess whether individual values (traditional, rational, survival, and self-expression) significantly influence voluntary membership among Europeans 50+. Specifically, we evaluated the extent to which these values interact with age (two groups: 50–64 and 65–79 years) and gender in predicting affiliation. Furthermore, since individual values cannot be studied independently of the socio-economic context of a country, we control the results for economic and macro-social factors at the country level (Gini index, GDP, and Welfare Systems).

It is expected that, individuals with high traditional and self-expressive values will report high ratios of organizational membership when compared to individuals with high secular and survival values (Hypothesis 1). As well, it is expected that, the 50–64 age group (vs. 65–79) will be more associated with voluntary organizations when they score high (vs. low) on traditional and self-expressive values (Hypothesis 2). Finally, it is expected that, men (vs. women) will be more associated with voluntary organizations when they score high (vs. low) on traditional and self-expressive values (Hypothesis 3).

Finally, because only a few researchers have analyzed the impact of these values on different types of voluntary membership among senior citizens, we conducted separate model tests for membership in voluntary social awareness, professional and political, educational and leisure, and religious organizations. We consider these aspects of the study to be exploratory.

## Materials and methods

Several data resources are used: (1) the Integrated Values Survey (IVS; 2005/09, 2010/14, and 2017/20), to select dependent variables (membership in a voluntary organization and categories), values (traditional/secular values and survival/self-expression values), and individual predictors (age, gender, educational level, income level, and marital status); (2) the Gini index (Eurostat, 2005/20); (3) Gross Domestic Product per capita (World Bank, 2005/20), which we used to obtain data at the national-level. The study focuses on European countries, as they are sufficiently homogeneous to make inferences from the results.

The sample consists of 31,985 individuals aged 50–79 years residing in twelve countries in Europe (Cyprus, Germany, the Netherlands, Poland, Romania, Slovenia, Spain, Sweden, Turkey, Ukraine, Russia, and Georgia), for three-time waves (2005/09, 2010/14 and 2017/20).

### Measures

#### Individual-level variables

Membership in voluntary organizations constitutes the dependent variable of this research. The IVS examines voluntary membership in eight types of organizations. The organizations are categorized into four groups (Gil-Lacruz M. et al., 2019; Sánchez-García et al., 2022): (1) Social Awareness (environmental, humanitarian, and charitable organizations); (2) Professional and Political (professional organizations, political parties, and labor unions); (3) Education and Leisure (sports and recreational clubs, arts, music, and educational organizations); and (4) Religion (religious organizations or churches). The variable of membership in voluntary organizations was coded as a dichotomous variable: “1” denotes that the individual belongs to the organization and ‘0’ indicates that the individual does not belong.

The IVS database contains a wide variety of sociodemographic variables: gender (Men and Women), age in two groups (Middle age: 50–64 and Old age: 65–79), educational level (Primary, Secondary, and Tertiary), income level (Low, Medium, and High) and marital status (Married, Single, Divorced, and Widowed). Furthermore, it includes two dimensions of values (Inglehart and Welzel, 2005): The Traditional-Secular Values Index and The Survival-Self-expressive Values Index.

The two dimensions were created following the protocol indicated by the World Values Survey created by Inglehart and Welzel (2005). The two values indices are created by performing a factor analysis on a set of ten indicators (importance of God, autonomy index, justification of abortion, national pride, respect for authority, happiness, justification of homosexuality, signing a petition, trust, and materialist/post-materialistic values index). The ten indicators used (five for each dimension) following the WVS procedure are selected for technical reasons, i.e., they appear in all three waves of the IVS. The positive pole of the first factor is rational, and the negative pole is traditional. The positive pole of the second factor is self-expression, and the negative pole is survival.

#### National-level variables

Countries are grouped by welfare systems (Sardinha, 2011; Sánchez-García et al., 2022): (1) Nordic: Sweden; (2) Continental: Germany, the Netherlands; (3) Mediterranean: Cyprus, Spain and Turkey; (4) Eastern: Poland, Romania, Slovenia, Ukraine, Russia, and Georgia. Geographic dummy variables were created for each country and for each welfare system. Dummy variables were also generated for each wave (2005–2009, 2010–2014, and 2017–2020). The set of dummy variables allows the calculation of geographic and temporal effects.

Finally, explanatory variables at the national level facilitate the comprehension of the differences between countries and welfare systems. Gross Domestic Product (GDP, constant prices adjusted to purchasing power 2017 US dollars) in per capita terms are included. Moreover, the Gini index is considered as an index of income inequality. The Gini coefficient is defined as the ratio between the cumulative percentages of the population, ordered by level of equivalized disposable income, and the cumulative percentage of the total equivalized disposable income they receive (EUROSTAT, 2022); high scores (100) mean “complete inequality,” where one individual has all the resources, and low scores (0) mean “complete equality,” where everyone has the same resources.

### Procedure

Data are analyzed using STATA 14 (Stata Corporation, College Station, TX, United States). The different characteristics at the individual and national level are analyzed using descriptive statistics. Table 1 shows the estimated frequencies.

**TABLE 1:** Variables (means and standard deviation by gender and age).

	Total		Female		Male		50–64		65–79	
	Mean (%)	SD	Mean (%)	SD	Mean (%)	SD	Mean (%)	SD	Mean (%)	SD
**Dependent variables**										
All categories	40.9	0.49	38.6	0.49	43.9	0.50	41.1	0.49	40.7	0.49
Social Awareness	11.9	0.32	11.9	0.32	11.9	0.32	11.6	0.32	12.3	0.33
Politics and profession	19.0	0.39	15.4	0.36	23.6	0.42	22.0	0.41	14.5	0.35
Leisure and education	19.9	0.40	17.6	0.38	22.9	0.42	19.9	0.40	20.0	0.40
Religion	19.4	0.39	20.1	0.40	18.5	0.39	17.2	0.38	22.6	41.8
**Independent variables**										
Age: 50–64	60.2	0.49	60.1	0.49	60.3	0.49	100	0.00	0.00	0.00
Age: 55–79	39.8	0.49	39.9	0.49	39.7	0.49	0.00	0.00	100	0.00
Female	55.9	0.50	100	0.00	0.00	0.000	55.9	0.50	56.0	0.50
Male	44.1	0.50	0.00	0.00	100	0.00	44.1	0.50	43.9	0.50
Primary studies	33.3	0.47	33.4	0.47	33.1	0.47	29.8	0.46	38.6	0.49
Secondary studies	36.1	0.48	35.4	0.48	37.2	0.48	40.2	0.49	30.1	0.46
Tertiary studies	12.7	0.33	11.9	0.32	13.8	0.34	13.7	0.34	11.3	0.32
Low income	37.8	0.48	42.2	0.49	32.4	0.47	33.8	0.47	43.9	0.49
Middle income	51.6	0.50	49.1	0.50	54.7	0.50	53.8	0.50	48.4	0.50
High income	10.5	0.31	8.7	0.28	12.8	0.33	12.4	0.33	7.7	0.27
Married	66.5	0.47	57. 6	0.49	77.7	0.42	71.4	0.45	59.1	0.49
Divorced	10.1	0.30	11.3	0.32	8.7	0.26	12.2	0.33	7.0	0.26
Widow	17.8	0.38	25.9	0.44	7.5	0.26	9.8	0.30	30.0	0.46
Single	5.5	0.22	5.1	0.22	6.1	0.24	6.7	0.25	3.8	0.19
Traditional/secular values	17.8	1.54	9.2	1.50	26.9	1.57	22.5	1.56	9.0	1.50
Survival/self-expression values	14.3	2.05	2.4	2.07	28.7	2.01	18.9	2.03	7.0	2.07

We have also included Macro Variables (GDP ppp and Gini index). Regional Dummy Variables (Nordic: Sweden; Continental: Germany, the Netherlands; Mediterranean: Cyprus, Spain, and Turkey; and Eastern: Poland, Romania, Slovenia, Ukraine, Russia, and Georgia) and Time Dummy Variables (Wave 1: 2005–2009; Wave 2: 2010–2014; and Wave 3: 2017–2020).

Multilevel regression models (xtmelogit) are indicated when there is a hierarchical structure in the levels of the data (individual and national), with a single dependent variable measured at the lowest level and a set of explanatory variables at each level (micro and macro). The study allows the simultaneous consideration of micro variables (values, age, gender, educational level, income level, and marital status), and macro variables (GDP, Gini index, and welfare systems) to explain the decision to membership in voluntary organizations.

The estimation analysis considers a mixed-effects multilevel regression model. Data are obtained from individuals nested within countries. The model is estimated as,


(1) $$V\,o\,l\,u\,n\,t\,e\,e\,r\,m\,e\,m\,b\,e\,r_{i\,j}=\beta_{0}+X_{i\,j}^{\prime }\,\beta_{j}+u_{j}+e_{i\,j}$$

For *j* = 1,…, 12 European countries, with data structured for *j*1,…, 12observations, where *X* includes *K* regressors (*K*−1variables and a constant) and the error term denoted by *e*_*ij*_*N*(0,σ^2^). The model incorporates the coefficients of the regressions for the intercept β_0_ and fixed effects β_*j*_ as explanatory variables for membership in a voluntary organization; and random effects *u*_*j*_to include cross-country variability.

Estimates are repeated for the four categories of volunteering (*f* = 1for Social Awareness *f* = 2for Politic and Professional *f* = 3for Leisure and Education; *f* = 4for Religion). The fact that the same person may belong to different volunteer organizations implies that the categories are not exclusive, thus the estimates were made independently,


(2) $$V\,o\,l\,u\,n\,t\,e\,e\,r\,m\,e\,m\,b\,e\,r_{f\,i\,j}=\beta_{0}+X_{i\,j}^{\prime }\,\beta_{f\,j}+u_{f\,j}+e_{f\,i\,j}$$

The objective is to achieve accurate estimates of the β coefficients. To ensure the robustness of the estimated parameters, the Wald test was used to test for any problems related to endogeneity. The results are analyzed and presented stepwise in three models. According to test Hypothesis 1, focused on the association between individual variables and membership in voluntary organizations, Model 1 contains only individual factors (age–middle and old age -, gender, marital status, educational level, income level and cultural values) and time variables (2005–2020). This model is also used to analyze the hypothesized direct relationship (Hypothesis 2) of values with volunteer membership.

Model 2 simultaneously estimates the two levels, (1) individuals within countries, and (2) differences between countries. This model incorporates country-level factors (GDP, Gini index, and welfare systems). Model 2 is justified by the results obtained in ANOVA, a statistical analysis that considers the dispersion of random effects according to groups of countries classified by welfare systems. The dispersion of random effects is greater between groups than within groups, so that the welfare system represents an appropriate country classification model.

Finally, to examine the hypothesized interactions (Hypothesis 3), Model 3 incorporates the interactions between individual variables and cultural values. This model explores the impact of the effect of values on the probability of membership as a function of age and gender.

## Results

### Preliminary analysis

To prevent technical problems related to the endogeneity of the variables, tests were performed using the Wald test to ensure the direction of the association between the values and the dependent membership variables. The estimated parameters of the variables that could cause endogeneity problems were not significant (*p* > 0.01).

### Descriptive analysis

The results in Table 1 show the descriptive statistics of the variables studied. Because most of these variables are dummy variables (1/0), their means as percentages (multiplied by 100) provide us their corresponding participant distribution. For instance, the mean of membership in voluntary organizations is 0.41, which means that 41% of the participants report belonging to voluntary associations. The total sample is equally distributed between women (39%) and men (44%), as well as between both age groups (50–64 and 65–79). The majority of citizens are married (66%), especially men (78%) and adults aged 50–64 (71%). Most of the people surveyed have secondary education (36%); men (37%) and adults aged 50–64 (40%) have higher levels of education than women (35%) and those aged 65–79 (30%). Taking into account economic conditions, 38% of senior citizens have low income, 52% middle income, and only 11% high income.

The results in Table 1 reveal that men (44%) adhere slightly more frequently to voluntary organizations than women (39%). However, the ratios of membership vary according to the type of organization. On the one hand, men are more likely to join political and professional (24%), educational and leisure (23%) associations than women (15 and 18%, respectively). On the other hand, women more frequently belong to associations related to religion (20%), compared to men (18%).

There are no differences between age groups in the frequencies of membership in voluntary organizations, in general. However, while middle-aged adults aged 50–64 (22%) are more frequent members of political and professional voluntary organizations than older adults (15%), adults aged 65–79 (23%) are more frequent members of religious associations than middle-aged adults (17%).

**FIGURE 1:**
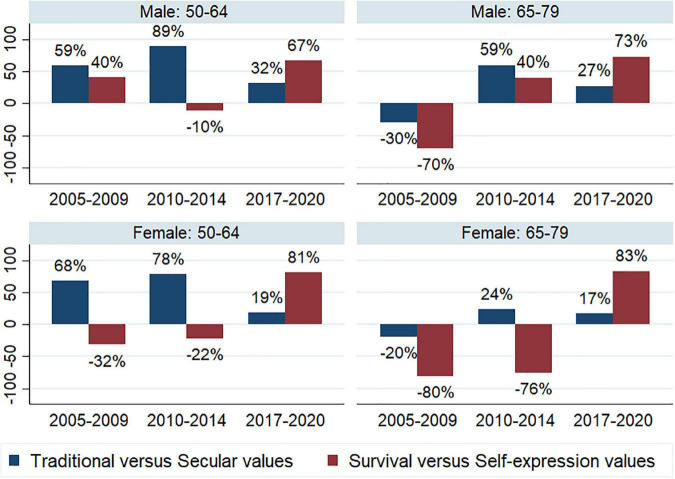
Values by gender and age, 2005/09, 2010/14, and 2017/20 (Data in percentages). Integrated Values Survey, own production.

In terms of individual values, men have more secular (27%) and self-expressive (29%) values than women (9 and 2%, respectively). However, these results vary according to age and time period considered, as shown in Figure 1. Positive percentages refer to secular and self-expression values, while negative percentages refer to traditional and survival values. Although an increase in rational values is observed from 2010 to 2014, a decrease in the same values is observed from 2014 to 2020, while an increase in progressive values is observed, regardless of age and gender. The shift from survival values to self-expression values is more prominent for older women than for older men. In recent years (2017 to 2020), men of any age group report being more rational than women, while women are more self-expressive than men.

### Multilevel analysis

Table 2 shows the probability of voluntary membership in general and by category (Model 1). As for the degree of variance of the random effect of Model 1 $\sigma_{u}^{2}$ is significant, this justifies the multilevel analysis of the dependent variable explained by the fixed and random effects. Sociodemographic and values variables are included in the fixed effects. Secondary and tertiary education level, and middle and high income level, are positively associated with membership in voluntary organizations. In addition, being married promotes membership in voluntary organizations in contrast to being divorced. These results are similar for each of the volunteer categories, except for religion, where primary education is more relevant for volunteer membership.

**TABLE 2:** Estimations of individual-level variables on volunteering: Model 1.

	All categories	Social awareness	Politic and professional	Leisure and education	Religion
Intercept	–0.60**	–2.69***	–2.16***	–2.08***	–1.62***
**Fixed effects**					
Age: 50–64	0.16***	–0.10**	0.51***	0.09***	–0.06
Age: 65–79^a^	–	–	–	–	–
Female	–0.08**	0.29***	–0.45***	–0.14***	0.22***
Male	–	–	–	–	–
Primary studies^a^	–	–	–	–	–
Secondary studies	0.12***	0.21***	0.12***	0.27***	–0.08**
Tertiary studies	0.67***	0.73***	0.69***	0.68***	0.08
Low income^a^	–	–	–	–	–
Middle income	0.30***	0.33***	0.48***	0.36***	0.06
High income	0.35***	0.32***	0.45***	0.42***	0.02
Married^a^	–	–	–	–	–
Divorced	–0.16***	–0.15**	–0.11*	–0.24***	–0.30***
Widow	–0.03	–0.11	–0.22***	–0.14**	0.04
Single	0.02	0.13	0.05	–0.10	0.01
Traditional/secular values	–0.10***	0.02	0.04***	0.00	–0.39***
Survival/self-expression values	0.13***	0.18***	0.20***	0.21***	–0.10***
Wave: 2005–2009	–0.40***	–0.48***	0.01	–0.46***	–0.40***
Wave: 2010–2014	–0.05	–0.33***	0.07	–0.25***	0.07
Wave: 2017–2020^a^	–	–	–	–	–
**Random effects**					
σ^2^	1.00	1.01	0.54	0.99	1.15
LR test (Prob[ > *X*^2^])	0.00	0.00	0.00	0.00	0.00
ICC	0.23	0.24	0.08	0.23	0.29
**Analysis of variance**					
Between groups	25627.37	17676.01	3475.06	25631.12	33442.39
Within groups	7337.84	15380.70	4448.27	8967.55	8850.29
Barlett’s test	0.00	0.00	0.00	0.00	0.00

Coefficients are reported. ICC: intra-class correlation index. ***, **, and * explanatory variables are statistically significant at 99, 95, and 90% levels.

^a^Variable of reference.

Men are more likely to belong to voluntary organizations than women, in general. Furthermore, there are differences depending on the type of activity. While men are more willing to become members of professional, political, educational and leisure organizations, women are more likely to be members of social awareness and religious organizations. With respect to age, middle-aged individuals are more likely to be volunteer members than older individuals. There are also variations by type of organization, with middle-aged individuals more frequently belonging to political and educational organizations, while older individuals are more inclined to be members of social awareness organizations.

The results found in Model 1 are congruent with Hypothesis 1. Hence, individuals with traditional and self-expressive values are more likely to be members of voluntary organizations than individuals with secular and survivalist values. Although it has not been hypothesized, this tendency varies according to the type of association. On the one hand, high traditional values positively influence voluntary membership in religious organizations, while high rational values are positively related to voluntary membership in professional and political associations. On the other hand, high self-expressive values favor adherence to social awareness, professional and political, and educational and leisure organizations; whereas survival values promote voluntary membership in religious associations.

Model 2 incorporates GDP, Gini index and welfare systems as fixed effects. The coefficients of the individual variables remain stable as an indicator of robustness, as shown in Table 3. As for the national variables, GDP per capita turns out to be a positive indicator of affiliation coefficients. The Gini index has a negative relationship with membership in voluntary organizations. This means that the greater the economic inequality in a country, the lower the probability of joining an association. With respect to welfare systems, the Nordic regime positively influences voluntary membership in general, compared to Eastern countries. However, residents of Eastern countries are more frequently associated with religious organizations than Nordic citizens. In Mediterranean countries there is a high probability of voluntary membership in political, professional, and religious organizations.

**TABLE 3:** Estimations of individual-level and national-level variables on volunteering: Model 2.

	All categories	Social awareness	Politic and professional	Leisure and education	Religion
Intercept_	–5.21***	–7.48***	–3.63***	–3.86***	–2.08*
**Fixed effects**					
Age: 50–64	0.17***	–0.09***	0.51***	0.09**	–0.06
Age: 65–79^a^	–	–	–	–	–
Female	–0.07**	0.29***	–0.45***	–0.14***	0.22***
Male^a^	–	–	–	–	–
Primary studies^a^	–	–	–	–	–
Secondary studies	0.12***	0.20***	0.12***	0.27***	–0.08**
Tertiary studies	0.68***	0.74***	0.69***	0.68***	0.08
Low income^a^	–	–	–	–	–
Middle income	0.30***	0.33***	0.48***	0.36***	0.06
High income	0.34***	0.31***	0.44***	0.42***	0.01
Married^a^	–	–	–	–	–
Divorced	–0.16***	–0.16**	–0.11*	–0.24***	–0.31***
Widow	–0.03	–0.11	–0.22***	–0.14**	0.04
Single	0.01	0.12**	0.05	–0.10	0.01
Traditional/secular Values^b^	–0.10***	0.02**	0.04***	0.01	–0.39***
Survival/self-expression values^b^	0.13***	0.18	0.20***	0.20***	–0.10***
Gini index	0.14***	0.13**	0.03**	0.02	0.05**
GDP	0.00***	0.00***	0.00***	0.00***	0.00*
Nordic^a^	–	–	–	–	–
Continental	–0.85	–0.46	–0.89	0.24	–0.68
Southern	–2.81**	–1.40	–1.02	–0.71	2.98***
East	–1.71	–1.26	–0.62	–0.49	2.09***
Wave: 2005–2009	–0.18***	–0.18*	0.16	–0.22***	–0.27***
Wave: 2010–2014	0.11**	–0.10	0.19*	–0.07	0.16***
Wave: 2017–2020^a^	–	–	–	–	–
**Random effects**					
σ^2^	1.00	1.10	0.46	0.45	0.72
LR test (Prob[ > *X*^2^])	0.00	0.00	0.00	0.00	0.00
ICC	0.23	0.27	0.06	0.06	0.13

Coefficients are reported. ICC: intra-class correlation index. ***, **, and * explanatory variables are statistically significant at 99, 95, and 90% levels.

^a^Variable of reference.

^b^The Wald endogeneity test was estimated. There is no empirical evidence of endogeneity.

The variance of the random effects is reduced in Model 2, that is, with the introduction of national data. Once the results have been controlled for individual and national variables, we are interested in testing whether the association between age and membership, as well as between gender and membership, is moderated by cultural structures. Hypotheses 2 and 3 focus on the effect of traditional and self-expressive values on the relationship between age and membership, on the one hand, and on the relationship between gender and membership, on the other hand.

As presented in Model 3 in Table 4, traditional and self-expressive values do not interact with age in associationism in general, thus Hypothesis 2 cannot be supported. The exploratory analyses for each type of organization show that traditional values do interact with age in religion. Consequently, middle-aged individuals (50–64) are more likely to associate with religious organizations when they have rational values. In addition, self-expression values increase the likelihood that older adults (65–79) associate with social awareness and education and leisure organizations. In addition, self-expression values increase the likelihood that middle-aged adults (50–64) participate in political and professional organizations.

**TABLE 4:** Estimates of the moderating effect of values on volunteering: Model 3 by age and gender.

	All categories	Social awareness	Politic and professional	Leisure and education	Religion
Intercept_	–5.19***	–7.37***	–3.60***	–3.70***	–2.10*
**Fixed effects**					
Age: 50–64	0.16***	–0.03	0.47***	0.16***	-0.06*
Age: 65–79^a^	–	–	–	–	–
Female	–0.09***	0.17***	–0.45***	–0.26***	0.24***
Male^a^	–	–	–	–	–
Primary studies^a^	–	–	–	–	–
Secondary studies	0.12***	0.19***	0.12***	0.27***	–0.08**
Tertiary studies	0.68***	0.74***	0.70***	0.68***	0.08
Low income^a^	–	–	–	–	–
Middle income	0.30***	0.33***	0.48***	0.36***	0.06
High income	0.34***	0.34***	0.45***	0.44***	0.01
Married^a^	–	–	–	–	–
Divorced	–0.16***	–0.16**	–0.11*	–0.24***	–0.31***
Widow	–0.03	–0.08	–0.22***	–0.11*	0.03
Single	0.01	0.12	0.04	–0.10	0.00
Traditional/secular values^b^	–0.15***	–0.05*	–0.01	–0.02	-0.45***
Survival/self-expression values^b^	0.12***	0.19***	0.18***	0.20***	–0.09***
Gini index	0.14***	0.13***	0.04**	0.01	0.05**
GDP	0.00***	0.00***	0.00***	0.00***	0.00*
Nordic^a^	–	–	–	–	–
Continental	–0.86	–0.46	–0.90*	0.25	–0.69
Southern	–2.84**	–1.38	–1.05*	–0.65	–3.00***
East	–1.73*	–1.23	–0.65	–0.43	2.11***
Age 50–64 × Traditional/secular	0.03	0.03	0.02	–0.01	0.05**
Age 50–64 × Survival/self-expression	0.02	–0.05**	0.04**	–0.05**	0.01
Female × Traditional/secular	0.06***	0.10***	0.07***	0.07***	0.04*
Female × Survival/self-expression	–0.00	0.04*	–0.01	0.07***	–0.03*
**Random effects**					
σ^2^	1.00	1.09	0.46	0.42	0.72
LR test (Prob[ > *X*^2^])	0.00	0.00	0.00	0.00	0.00
ICC	0.23	0.26	0.06	0.05	0.14

Coefficients are reported. Estimations have also been controlled by waves. These results have been omitted to improve presentation, but they are available on request. ICC, intra-class correlation index. ***, **, and * explanatory variables are statistically significant at 99, 95, and 90% levels.

^a^Variable of reference.

^b^The Wald endogeneity test was estimated. There is no empirical evidence of endogeneity.

Finally, traditional values do interact with gender, but not in the way we expected according to Hypothesis 3. Women with high secular values affiliate more frequently with voluntary organizations than men. However, significant interactions are found in the exploratory analyses in the categories. Women who score high on rational values are volunteer members in each type of organization. In addition, self-expressive values increase the willingness of women to belong to social awareness, education, and leisure organizations; while survival values increase the adherence of women to be members in religious associations.

## Discussion

The results of this study provide new evidence on the determinants of membership of voluntary organizations among 50+ years of age citizens in Europe, following the model of the volunteering process with respect to its “antecedents” (Snyder and Omoto, 1992): (1) examines differences in voluntary membership between women and men in middle age and old age with integrated data at two levels: individual and national, (2) highlights the influence of individual values in explaining voluntary membership in general (Inglehart and Welzel, 2005) and in different types of associations, (3) shows how values can explain age and gender differences in voluntary membership. The findings are intended to further the understanding of voluntary membership in order to promote this activity and avoid a state of “mental retirement” that can lead to early inactivity, with negative health consequences.

Volunteering by older people is a way of being active, as well as an opportunity to work with others when work and family roles have been fulfilled (Principi et al., 2014). The Theory of Activity proposes that, after retirement, older people engage in volunteering activities as a means of maintaining a positive sense of self and feeling meaningful and productive (Lemon et al., 1972; Havighurst et al., 1996). In this sense, volunteering can play an important role for older people by protecting them from the negative effects of retirement, physical decline, inactivity and social isolation (Fiorillo and Nappo, 2014; Coll-Planas et al., 2017). Some studies have shown that older people who volunteer report better health status than older people who do not volunteer (Fancourt and Steptoe, 2018; Huo et al., 2021; Moyano-Díaz and Mendoza-Llanos, 2021).

Despite the beneficial effects of volunteering on older people, such as improved physical function, self-reported health and life satisfaction (Gil-Lacruz M. et al., 2019), most older people engage in volunteer activities only after being asked, while a minority actively seek out volunteering opportunities (Chen and Morrow-Howell, 2015). Therefore, it is relevant to know the individual characteristics that promote the drive for this type of behavior. Undoubtedly, knowing the determinants of voluntary membership will help the recruitment of older people (Heist et al., 2019).

The study data show that middle-aged, male, with higher-income level, higher educational level and married adults are more likely to belong to voluntary organizations. In contrast, older, female, with lower educational level and divorced adults are less willing to enroll in voluntary organizations. These results are consistent with the scientific literature (Gil-Lacruz et al., 2017) and with theories of volunteering at the individual level (Wilson, 2000). Consequently, the ability to be a member of an association is determined by the resources that it possesses.

Educational level and income level are the most consistent variables in predicting membership in voluntary organizations in line with other studies (Prouteau and Wolff, 2004; Gil-Lacruz and Marcuello, 2013). Resource theorists assume that individuals with greater resources tend to be more likely to adhere to voluntary associations (Wilson and Musick, 1999; Musick et al., 2000). The theorists argue that “the desire to do good is more or less distributed, but the resources to fulfill that desire are not” (Wilson and Musick, 1999, p. 244). Hence, human capital is seen as a determinant of volunteerism. There have been several interpretations of the positive relationship between educational level and volunteerism. For instance, people with higher levels of education have highly developed organizational and communication skills (Brady et al., 1995), a strong sense of civic responsibility and understanding of community issues (Wilson and Musick, 1997), and extensive social networks that increase their chances of being asked to volunteer (Brady et al., 1999).

In addition, income has also been associated with volunteering. Two explanations can be considered in terms of costs and benefits (Musick and Wilson, 2007, p. 127). The first is the Opportunity Cost Theory, which assumes that those with higher incomes spend less time volunteering because of the loss of wages by doing volunteering. The second is the Resource Theory, which argues that people with higher incomes spend more time volunteering because their abundant resources allow them to volunteer. Since our results report a positive relationship between income and membership in voluntary organizations, we argue for the latter.

In terms of age, the lowest membership ratios are found among older people of unemployed age (65–79 years). The results contrast with the Rational Choice Theory, which predicts an increase in voluntary membership at retirement age because more time is available. Similarly, the Exchange Theory assumes that retirees seek to engage in voluntary work to replace the benefits formally derived from paid employment (Fischer et al., 1991; Midlarsky and Kahana, 1994). However, both theories agree with the discourse that the highest level of involvement in voluntary organizations occurs in middle age (Menchik and Weisbrod, 1987).

Following the analysis of sociodemographic variables, the results show that, in general, men are more frequently adhered to voluntary organizations than women, which is consistent with the literature (Gil-Lacruz A. I. et al., 2019). One possible explanation for this is that men possess higher resources than women; for example, men possess higher educational level and income level than women. The fact that women and older people report the same results is in consonance with the Life-Cycle Theory, in which membership ratios to a voluntary organization decrease in old age, especially among women (Gallagher, 1994).

However, these results vary according to the type of activity (Sánchez-García et al., 2022), with middle-aged men (50–64) being more likely to associate with professional, political and educational organizations; whereas older women (65–79) are more likely to associate with social awareness and religious organizations. In this sense, socio-economic determinants are insufficient to draw firm conclusions to explain gender and age differences (Wiepking et al., 2022). According to the Process Model of Volunteering (Snyder and Omoto, 1992; Wilson, 2012), motivations, beliefs and values are significant as well.

In relation to the above, the cultural and social climate of a society co-determines the attitudes of individuals, as well as values, and behaviors (Halman and Gelissen, 2019; Akaliyski et al., 2021; Spitsyna and Koval, 2022). The results reveal that traditional and self-expressive values encourage adherence to voluntary organizations. This is related to the theories of modernism (Inglehart and Baker, 2000; Dekker and Halman, 2003; Welzel, 2021). According to modernism, volunteerism and its membership are associated with the degree of industrialization of a country and the emergence of the knowledge society. Thus, in an increasingly developed society the shift from traditional to secular values leads to lower ratios of civic activism, while the shift from survival to self-expressive values leads to higher levels of activism in general.

Some authors argue that due to modernization processes such as differentiation and specialization, each area of life has specific values (Durkheim, 1964; Fox, 2016). Therefore, it would be logical to think that each domain of voluntary membership is influenced by certain values. It is found that, membership in social awareness organizations, and educational and leisure time organizations are influenced by self-expression values. Professional and political organizations are influenced by self-expression and secular values. And the religious by traditional and survival values.

The strong influence of self-expressive values can be explained by the fact that, in societies where people have these types of values, opportunities for freedom of association and open discussion are unlimited (Musick and Wilson, 2007). In addition, the influence of traditional values on voluntary membership in religious associations might be due to the reason that, in these types of organizations, people have simpler and more direct relationships with others, and therefore tend to attend more frequently when they are called upon (Luria et al., 2017).

In terms of age, the frequency of affiliation of middle-aged individuals (50–64) to political and professional organizations is stronger when they possess self-expressive values. These values even lead older adults (65–79) to belong to educational and leisure organizations, as well as social awareness organizations. These findings show that values are not specific to one age group but can be found in different generations (Wollebæk and Selle, 2003), and shape behavior in favor of voluntary membership.

With respect to gender, although men express more willingness to be affiliated to volunteer organizations than women, when women possess rational values they are more often involved than men in all categories of volunteering. In addition, self-expression values help women to participate in political and educational organizations, which have often been characterized by a male presence (Gil-Lacruz A. I. et al., 2019; Sánchez-García et al., 2022). One reason is that self-expressive values promote altruism (Welzel, 2021), a characteristic of women (Wilson, 2000), and that self-expressive societies support gender equality (Inglehart, 2007).

According to Salamon and Anheier’s (1998) Theory of Social Origins, the influence of attitudes, beliefs and values cannot be considered in isolation from the economic and temporal context of a country. The findings indicate that economic inequality mobilizes individuals to participated in voluntary organizations, probably in seeking social change. In addition, citizens of Sweden report higher membership ratios in voluntary organizations compared to Mediterranean countries. Nordic governments are characterized by limited private welfare provision, which encourages people to be active (Esping-Andersen, 1990). Moreover, Nordic countries provide more care and help services (Bergquist et al., 2018; Costa-Font, 2019), which may encourage affiliation among older people, and women, by reducing the need to be caregivers.

### Limitations

The absence of Anglo-Saxon countries evaluating voluntary membership in the WVS prevents the analysis of the Anglo-Saxon welfare system, which limits the generalization of the results to the European level. In line with the above, data are only available for Sweden within the Nordic regime.

Future studies could consider a wide range of values, such as those related to Achievement, Benevolence, Power, Universalism, Individuality, Hedonism, Tradition, Security, Conformity, and Stimulation (Schwartz, 1992) or those related to Distance to Power, Individualism versus Collectivism, Uncertainty Avoidance, Masculinity versus Femininity, Long-Term Orientation and Complacency versus Moderation (Hofstede, 1991).

### Practical implications

Retirement is a long-awaited life transition for many older people, although in some cases the exit from the labor force implies a loss of meaningful social interactions and sense of productivity (Jolles et al., 2022). In this sense, membership in volunteer organizations mitigates these negative effects and even improves the subjective well-being of unemployed older people (Gil-Lacruz M. et al., 2019; Chung et al., 2020). Given the synergy arising from social cooperation and well-being, more attention and consideration should be given to volunteer membership. It should be recognized as a key element in the development of social cohesion and have much more visibility in society (Deloitte Consulting, 2012).

Since values influence voluntary membership ratios, organizations and policy makers could improve the choice of terms to refer to older people in equality (Vauclair et al., 2016). Moreover, organizations could provide clearer information about the principles, values and objectives of the association so that seniors can check whether they are in line with their values. In this way, voluntary organizations could improve recruitment and retention strategies for seniors. At the societal level, it is necessary improve opportunities for equal access to a voluntary organization across all age groups (Swift et al., 2017). This involves ensuring pension systems that provide economic security for older people, ensuring equal access to medical and social care, investing in age-friendly structures, and offering educational opportunities throughout life (Fasel et al., 2021).

## Conclusion

In conclusion, the present findings contribute to the existing literature on the determinants of membership in voluntary organizations among the older adults. It provides important insight into the importance of personal values, demonstrating that those who hold traditional and self-expressive values adhere more frequently to voluntary organizations. The Theory of Reasoned Action (Fishbein and Ajzen, 1975) already demonstrated that values are one of the components that can explain human behavior. People with different values and norms will be more attracted to different organizations, and a distinction of types of volunteering seems to be a condition for a better understanding of values at the individual level. Specifically, self-expressive and rational values are most likely to encourage membership of older women in voluntary associations, even in those that traditionally have been male-dominated (e.g., political and professional). The fact that these values lead women to become volunteer members more often could have an impact on society in advancing equality (Wiepking et al., 2022). “Culture includes what we think, how we act and what we own” (Macionis and Plummer, 1998, p. 98). Therefore, resources are not the only determinants of membership in voluntary organizations; personal values may also affect this prosocial behavior among the older adults.

## Data availability statement

Publicly available datasets were analyzed in this study. This data can be found here: Micro-data from the Integrated Values Survey (2005–2009; 2010–2014, and 2017–2020), and macrodata from World Bank (2020) and EUROSTAT (2022).

## Author contributions

JS-G: conceptualization, investigation, methodology, formal analysis, visualization and data presentation, writing–original and final drafts, review, and editing. JS-G and AG-L: data collection and analysis. JS-G and MG-L: review, writing, and editing of the conceptual framework. JS-G, AV-T, DM-T, and MM: review and editing. All authors approved the submitted version.
